# The interactions between interleukin-1 family genes: *IL1A*, *IL1B*, *IL1RN*, and obesity parameters

**DOI:** 10.1186/s12864-021-08258-x

**Published:** 2022-02-09

**Authors:** Ewelina Maculewicz, Bożena Antkowiak, Oktawiusz Antkowiak, Anna Borecka, Andrzej Mastalerz, Agata Leońska-Duniec, Kinga Humińska-Lisowska, Monika Michałowska-Sawczyn, Aleksandra Garbacz, Katarzyna Lorenz, Ewa Szarska, Łukasz Dziuda, Anna Cywińska, Paweł Cięszczyk

**Affiliations:** 1 Faculty of Physical Education, Jozef Pilsudski University of Physical Education in Warsaw, 00-809 Warsaw, Poland; 2 Military Institute of Hygiene and Epidemiology, 01-163 Warsaw, Poland; 3 Faculty of Physical Education, Gdansk University of Physical Education and Sport, 80-336 Gdansk, Poland; 4 Warsaw University of Life Sciences - SGGW, 02-787 Warsaw, Poland; 5 Military Institute of Aviation Medicine, 01-755 Warsaw, Poland; 6 Faculty of Biological and Veterinary Sciences, Nicolaus Copernicus University in Torun, 87-100 Torun, Poland

**Keywords:** Genetic polymorphisms, *IL1A*, *IL1B*, *IL1RN*, Body mass index, Fat percentage

## Abstract

**Background:**

Obesity has been recognized as a worldwide growing problem, producing many pathologies including the promotion of “proinflammatory state.” The etiology of human obesity is still only partially understood; however, the genetic background has been proved. Its nature is complex, and currently, it appears that the combined effects of the interactions among multiple genes should receive more attention. Due to the fact that obesity promotes proinflammatory conditions, in this study, we investigated the genetic polymorphism of IL-1 family genes in healthy people with normal and elevated body mass index (BMI) and fat %.

**Results:**

The single-nucleotide polymorphisms (SNPs) within the *IL1A* -889C > T (rs1800587), *IL1B* + 3954 T > C (rs1143634), and *IL1RN* -87G > A (rs2234677) genes alone were associated neither with BMI nor fat % values in tested group. The associations between SNP–SNP interaction and BMI for the *IL1B* × *IL1RN* interactions were significant for dominant model (*p* = 0.02) and codominant model (*p* = 0.03). The same SNP-SNP interaction (*IL1B* × *IL1RN*) was associated also with fat % for codominant (*p* = 0.01) and recessive (*p* = 0.002) models.

**Conclusions:**

This study further confirmed that IL-1 family genes are involved in genetic background of obesity. It has been shown that interaction *IL1B* × *IL1RN* was associated with both BMI and fat % with rare T allele protecting form higher values. Thus, even if certain polymorphisms in single genes of IL-1 family cannot be defined as related to obesity in examined population, the genetic interrelationships should be analyzed.

## Background

In 20th century, obesity has been understood as a health problem and has reached epidemic proportions globally. According to the World Health Organization (WHO) data, it contributes to at least 2.8 million deaths each year. Once associated with high-income countries, obesity is now also prevalent in low- and middle-income countries, among people of any age [1]. From 1975 to 2016, an alarming, over fourfold growth (from 4 to 18%) in the prevalence of overweight or obesity was reported also in children and adolescents up to 19 years old. In 2017, over 4 million overweight or obese people died [2], and for the first time in human history, it was shown that the number of obese people worldwide exceeded underweight ones [3]. Thus, obesity represents a serious burden for public health.

Currently, the understanding of obesity is wider than only overweight and involves more aspects that make obesity a complex disease with multiple etiology and background. According to Rajender et al. (2014), human obesity arises from the combined effects of the interactions among multiple genes, environmental factors, and behavior. Thus, the management and prevention of this problem is especially challenging [4].

The etiology of obesity is complex and still not completely understood, but the role of environmental and genetic factors, as well as individual habits are widely accepted. The genetic background of obesity was proposed years ago, and it has been shown that the heritability of body mass index (BMI) ranges from 40 to 70% [5], and multiple genes and associations are involved. BMI is the most frequently used proxy for body composition; however, it is known that this value underestimates adiposity (body fat percentage). Since 1980, the American Army has implemented an Army Weight Control Program (AWCP), which originally was based on the soldier’s BMI but now body fat percentage (fat standard) is considered more important. Both values are tracked for soldiers and limits are implemented for military services [6].

Obesity is understood not only as abnormally high body weight (and so that high BMI) but also as a disease resulting in abnormal metabolic and immune functions. It has been shown that in obese individuals, the proinflammatory state, expressed as chronic, low-grade inflammation, is created, and may result in numerous inflammatory conditions [7–9]. It has been proved that the adipose tissue acts as a potent endocrine organ and a source of cytokines [7, 10–12], as indicated by increased concentrations of IL-6 and IL-1 in obese patients [13, 14].

The IL-1 is a cytokine family that has been shown to play a key role in multiple innate and acquired immune processes, including inflammation, fibrosis, autoimmune responses, vascular diseases, neoplasia, and others [7, 15, 16]. The IL-1 family consists of 11 members, including 7 with proinflammatory activity (IL-1α, IL-1β, IL-18, IL-33, IL-36α, IL-36β, and IL-36γ) and 4 with antagonistic (IL-1Ra, IL-36Ra, and IL-38) or anti-inflammatory (IL-37) properties. The IL-1 receptor family consists of 10 members: IL-1R1, IL-1R2, IL-1R accessory protein (IL-1RAcP), IL-18Rα, IL-18Rβ, ST2 (IL-33R), IL-36R (previously IL-1Rrp2), single-Ig IL-1R-related molecule (SIGIRR or TIR8), three-Ig-domain-containing IL-1R related 2 (TIGIRR-2 or IL-1RAPLI), and TIGIRR-1 (IL-1RAPL2) [7, 15, 17]. The balance between agonists and antagonists has been reported as being crucial for numerous inflammatory diseases, and the role of IL-1α, IL-1β, and IL-1Ra has been discussed the most frequently [7, 16]. The IL-1 family (except IL-18 and IL-33) is encoded as a cluster located in chromosome 2 (including *IL1A, IL1B*, and *IL1RN*) in locus 2q13-21 with three polymorphic genes [7, 16, 18]. Genetic polymorphisms of these genes have been indicated as potentially important in inflammatory diseases, including inflammatory bowel diseases, alopecia areata, psoriasis, lupus erythematosus, Alzheimer’s disease, joint degeneration, and metabolic syndrome, as well as obesity [7, 19–23].

Taking into account the role of IL-1 cytokines and receptors in the inflammatory response and the fact that proinflammatory/anti-inflammatory balance is clearly dysregulated in obese people, we hypothesized that variations and associations among IL-1 family genes may be important as promoting obesity. Thus, the aim of this study was to investigate genetic polymorphisms and interrelationships among genes of IL-1 family in healthy people (without inflammatory disorders) in the context of commonly used obesity measurements: BMI and the percentage of fat mass.

## Results

### Participants

The study population has been selected to maintain maximal homogeneity and to eliminate as many environmental factors as possible. Therefore we have selected 101 military professionals, which lived together, ate the same food, and had very similar daily schedule. For each analysis, the participants were divided into 2 groups differentiated by either BMI or fat % (Table 1). The groups based on BMI involved 76 control (CON_BMI_) individuals with BMI values between 20.0 and 25.0 and 25 overweight (OVER_BMI_) ones, with BMI > 25.0. The groups based on fat % were composed of 87 control (CON_Fat_) subjects with fat % below 20.0% and 14 overweight (OVER_Fat_) ones with fat % over 20.0%.

**Table 1:** Body parameters (expressed as mean ± SD) in the analyzed groups

Group	Age	Height	Weight	Visceral tissue index	Fat %	BMI
OVER_BMI_ (*n* = 25)	21,8 ± 1,64	180,8 ± 7,34	89,1 ± 8,63***	5,1 ± 1,63***	21,0 ± 3,06***	27,2 ± 1,85***
CON_BMI_ (*n* = 76)	21,9 ± 1,61	180,1 ± 6,39	75,3 ± 6,70***	2,1 ± 1,29***	14,5 ± 2,68***	23,2 ± 1,26***
OVER_Fat_ (*n* = 14)	21,4 ± 1,6	180,4 ± 8,4	89,9 ± 10,5***	5,8 ± 1,8***	22,9 ± 2,9***	27,6 ± 2,4***
CON_Fat_ (*n* = 87)	21,9 ± 1,6	180,3 ± 6,3	77,0 ± 7,8***	2,4 ± 1,4***	15,1 ± 2,9***	23,7 ± 1,7***

***p < 0.001 (Student’s *t*-test)

### Genetic analyses for the groups

The measured frequencies of the *IL1A* rs1800587, *IL1B* rs1143634, and *IL1RN* rs2234677 genotypes did not differ significantly from the Hardy-Weinberg equilibrium expectations in the OVER_BMI_ and OVER_Fat_ groups (*p* values 0.55–1 and 0.35–1, respectively). For the CON_BMI_ and CON_Fat_ groups, genotype *IL1B* rs1143634 and *IL1RN* rs2234677 frequencies were not significantly different from the Hardy-Weinberg equilibrium expectations (*p* values ranged 0.28–0.56 and 0.19–0.59, respectively), but for the *IL1A* rs1800587 genotype, where the *p* value was 0.01, expectations were fulfilled neither for CON_BMI_ nor CON_Fat_ group. For whole group, consisting of either CON_BMI_ and OVER_BMI_, or CON_Fat_ and OVER_Fat_, frequencies of the *IL1B* rs1143634 and *IL1RN* rs2234677 genotypes did not differ significantly from the Hardy-Weinberg equilibrium expectations (*p* values ranged from 0.32 to 0.81), and for the *IL1A* rs1800587 genotype, the expectations were not fulfilled (*p* = 0.003) (Table 2).

**Table 2:** Frequencies for *ILA*, *ILB*, and *ILRN* genotypes in OVER, CON, and ALL groups, probabilities that the genotype frequencies do not differ from Hardy-Weinberg expectations (HWE) and minor allele frequency (MAF)

SNP	Genotype	OVER ***N*** (%)	HWE ***p***-value	CON ***N*** (%)	HWE ***p***-value	ALL ***N*** (%)	HWE ***p***-value	MAF (%)
**BMI groups**								
***IL1A*** **rs1800587**	C/C	16 (64)	0.55	40 (52.6)	0.01	56 (55.4)	0.003	Allele T (22.3)
	T/C	9 (36)		36 (47.4)		45 (44.6)		
	T/T	0 (0)		0 (0)		0 (0)		
***IL1B*** **rs1143634**	C/C	11 (44)	1	40 (52.6)	0.56	51 (50.5)	0.81	Allele T (28.2)
	T/C	11 (44)		32 (42.1)		43 (42.6)		
	T/T	3 (12)		4 (5.3)		7 (6.9)		
***IL1RN*** **rs2234677**	G/G	17 (68)	1	35 (46.0)	0.28	52 (51.5)	0.32	Allele A (26.7)
	A/G	7 (28)		37 (48.7)		44 (43.6)		
	A/A	1 (4)		4 (5.3)		5 (4.9)		
**Fat % groups**								
***IL1A*** **rs1800587**	C/C	9 (64.3)	1	47 (54.0)	0.01	56 (55.5)	0.003	Allele T (22.3)
	T/C	5 (35.7)		40 (46.0)		45 (44.5)		
	T/T	0 (0)		0 (0)		0 (0)		
***IL1B*** **rs1143634**	C/C	7 (50.0)	0.55	44 (50.6)	0.59	51 (50.5)	0.81	Allele T (28.2)
	T/C	5 (35.7)		38 (43.7)		43 (42.6)		
	T/T	2 (14.3)		5 (5.7)		7 (6.9)		
***IL1RN*** **rs2234677**	G/G	10 (71.4)	0.35	42 (48.3)	0.19	52 (51.5)	0.32	Allele A (26.7)
	A/G	3 (21.5)		41 (47.1)		44 (43.5)		
	A/A	1 (7.1)		4 (5.60)		5 (5.0)		

Table 3 summarizes the results of the association analysis of SNPs in the *IL1A*, *IL1B*, and *IL1RN* genes and BMI values. *IL1A* polymorphism rs1800587 was analyzed only in codominant model due to the lack of AA genotype. The *IL1A* rs1800587, *IL1RN* rs2234677, and *IL1B* rs1143634 genotypes were not related with BMI value (Table 3).

**Table 3:** Analysis of *IL1A* (rs1800587), *IL1B* (rs1143634), *IL1RN* (rs2234677) polymorphisms in relation to BMI. Due to the lack of AA genotype, only codominant model was analyzed (rs1800587). OR—odds ratio; CI—confidence interval; AIC—the Akaike information criterion

		OVER_**BMI**_ (***n*** = 25)	%	CON_**BMI**_ (***n*** = 76)	%	OR	95 %CI		***p***-value	AIC
*IL1A* rs1800587										
**Codominant**	C/C	16	64.0	40	52.6	1.00			0.32	116
	T/C	9	36.0	36	47.4	0.62	0.25	1.59		
*IL1B* rs1143634										
**Codominant**	C/C	11	44.0	40	52.6	1.00			0.50	117.6
	T/C	11	44.0	32	42.1	1.25	0.48	3.25		
	T/T	3	12.0	4	5.3	2.73	0.53	14.04		
**Dominant**	C/C	11	44.0	40	52.6	1.00			0.45	116.5
	T/C-T/T	14	56.0	36	47.4	1.41	0.57	3.51		
**Recessive**	C/C-T/C	22	88.0	72	94.7	1.00			0.28	115.9
	T/T	3	12.0	4	5.3	2.45	0.51	11.81		
**Overdominant**	C/C-T/T	14	56.0	44	57.9	1.00			0.87	117.0
	T/C	11	44.0	32	42.1	1.08	0.43	2.69		
*IL1RN* rs2234677										
**Codominant**	G/G	17	68.0	35	46.1	1.00			0.15	115.3
	A/G	7	28.0	37	48.7	0.39	0.14	1.05		
	A/A	1	4.0	4	5.03	0.51	0.05	4.97		
**Dominant**	G/G	17	68.0	35	46.1	1.00			0.05	113.3
	A/G-A/A	8	32.0	41	53.9	0.40	0.15	1.04		
**Recessive**	G/G-A/G	24	96.0	72	94.7	1.00			0.80	117.0
	A/A	1	4.0	4	5.03	0.75	0.08	7.04		
**Overdominant**	G/G-A/A	18	72.0	39	51.3	1.00			0.07	113.7
	A/G	7	28.0	37	48.7	0.41	0.15	1.09		

Gene–Gene interactions *IL1B* rs1143634 × *IL1A* rs1800587, *IL1RN* rs2234677 × *IL1A* rs1800587 and *IL1B* rs1143634 × *IL1RN* rs2234677 (only pairwise interactions were considered) were investigated for the same genetic models as for single-gene analyses (except the overdominant model). The frequency of the remaining genotypes did not differ significantly between groups. No association was found between SNP–SNP interaction and BMI for the *IL1B* rs1143634 × *IL1A* rs1800587 and *IL1RN* rs2234677 × *IL1A* rs1800587 interactions in the dominant and the codominant models, and the recessive model was not applied for these interactions.

A significant association was found between SNP–SNP interaction and BMI for the *IL1B* rs1143634 × *IL1RN* rs2234677 interaction in the dominant model, *p* = 0.02 and the codominant model, *p* = 0.03. For both models, the odds of being OVER_BMI_ for TC x GG was about 16 times lower than for CCxGG (Fisher test, *p* = 0.005). No association was found between SNP–SNP interaction and BMI for the *IL1B* rs1143634 × *IL1RN* rs2234677 interaction in the recessive model (Table 4). However, the odds of being OVER_BMI_ for TC x GG-AG was 3.6 times lower than for CCxGG-AG (Fisher test, *p* = 0.025).

**Table 4:** Analysis of *IL1B* (rs1143634) *× IL1RN* (rs2234677) interaction in relation to BMI (dominant model). OR—odds ratio; CI—confidence interval, NA – not applicable

*IL1RN (*rs2234677)																	
Dominant model																	
		G/G								A/G-A/A							
*IL1B*(rs1143634)		**OVER**_**BMI**_		**CON**_**BMI**_	**OR**		**95% CI**			**OVER**_**BMI**_	**CON**_**BMI**_	**OR**			**95% CI**		***p***
	C/C	10		15	1.00		NA	NA		7	20	0.52			0.16	1.70	**0.02**
	T/C	1		24	**0.06**		**0.01**	**0.54**		6	13	0.69			0.20	2.43	
	T/T	0		1	0.00		0.00	NA		1	3	0.50			0.05	5.51	
Codominant model																	
		G/G					A/G					A/A					
		**OVER**_**BMI**_	**CON**_**BMI**_	**OR**	**95% CI**		**OVER**_**BMI**_	**CON**_**BMI**_	**OR**	**95% CI**		**OVER**_**BMI**_	**CON**_**BMI**_	**OR**	**95% CI**		***p***
	C/C	10	15	1.00	NA	NA	6	17	0.53	0.16	1.81	1	3	0.5	0.05	5.51	**0.03**
	T/C	1	24	**0.06**	**0.01**	**0.54**	4	12	0.50	0.13	2.00	2	1	3.0	0.24	37.67	
	T/T	0	1	0.00	0.00	NA	1	3	0.50	0.05	5.51	0	0	NA	NA	NA	
Recessive model																	
		G/G-A/G								A/A							
		**OVER**_**BMI**_	**CON**_**BMI**_		**OR**		**95% CI**			**OVER**_**BMI**_	**CON**_**BMI**_		**OR**		**95% CI**		***p***
	C/C	16	32		1.00		NA	NA		1	3		0.67		0.06	6.93	0.07
	T/C	5	36		**0.28**		**0.09**	**0.84**		2	1		4.00		0.34	47.50	
	T/T	1	4		0.50		0.05	4.85		0	0		NA		NA	NA	

The *IL1A* rs1800587, *IL1RN* rs2234677, and *IL1B* rs1143634 genotypes were not related with fat %. Pairwise gene-gene interactions with fat % were investigated for the same genetic models as for BMI in single gene analyses (except overdominant model). A significant association was found between the SNP-SNP interaction and OVER_Fat_ for the *IL1B rs1143634* x *IL1RN* rs2234677 interaction in the codominant model, *p* = 0.01 and the recessive model, *p* = 0.002 (Table 5).

**Table 5:** Analysis of the *IL1B(*rs1143634) x *IL1RN* (rs2234677) interaction in relation to fat % (codominant model. OR—odds ratio, CI—confidence intervals, NA—not applicable

*IL1RN* (rs2234677)																	
Codominant model																	
*IL1B* (rs1143634)		G/G					A/G					A/A					
		**OVER**_**Fat**_	**CON**_**Fat**_	**OR**	**95% CI**		**OVER**_**Fat**_	**CON**_**Fat**_	**OR**	**95% CI**		**OVER**_**Fat**_	**CON**_**Fat**_	**OR**	**95% CI**		***p***
	C/C	6	19	1.00	NA	NA	4	19	**0.67**	**0.16**	**2.75**	0	4	0.00	0.00	NA	**0.01**
	T/C	1	24	**0.13**	**0.01**	**1.19**	0	16	0.00	0.00	NA	2	1	6.33	0.48	82.75	
	T/T	0	1	0.00	0.00	NA	1	3	1.06	0.09	12.14	0	0	NA	NA	NA	
Recessive model																	
		G/G-A/G							A/A								
		**OVER**_**Fat**_	**CON**_**Fat**_		**OR**		**95% CI**		**OVER**_**Fat**_	**CON**_**Fat**_		**OR**		**95% CI**			***p***
	C/C	10	38		1.00		NA	NA	0	4		0.00		0.00	NA		**0.002**
	T/C	1	40		**0.10**		**0.01**	**0.78**	2	1		7.60		0.62	92.54		
	T/T	1	4		0.95		0.10	9.47	0	0		NA		NA	NA		

The odds of being OVER_Fat_ for T/C x G/G (*ILB x IL RN*) was over 7 times lower than for reference combination C/C x G/G (*ILB x ILRN*). Similarly, the odd of being OVER_Fat_ for C/C x A/G (*ILB x ILRN*) was 1.5 times lower than for C/C x G/G (*ILB x ILRN*). All individuals representming T/C x A/G and C/C x A/A (*ILB x ILRN*) were in CON_Fat_ group (Fisher test, *p* = 0.09). The odds of being OVER_Fat_ for T/C x G/G-A/G (*ILB x ILRN*) was 10 times lower than for reference combination C/C x G/G - A/G (*ILB x ILRN*). All participants representing C/C x A/A (*ILB x ILRN)* were in CON_Fat_ group (Table 5, Fisher test, *p* = 0.01).

**Fig. 1:**
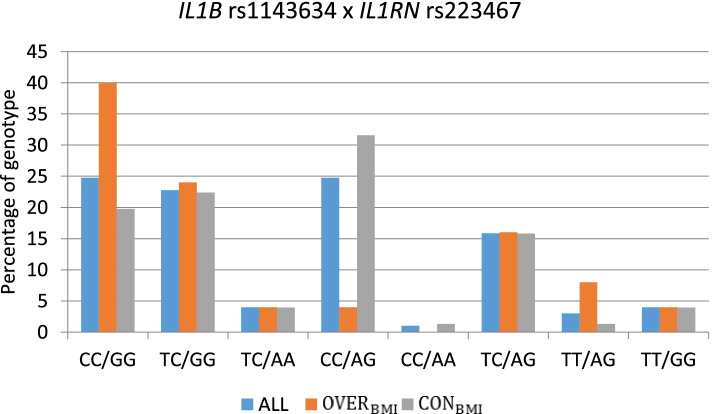
Respective genotype percentages in the examined BMI groups

**Fig. 2:**
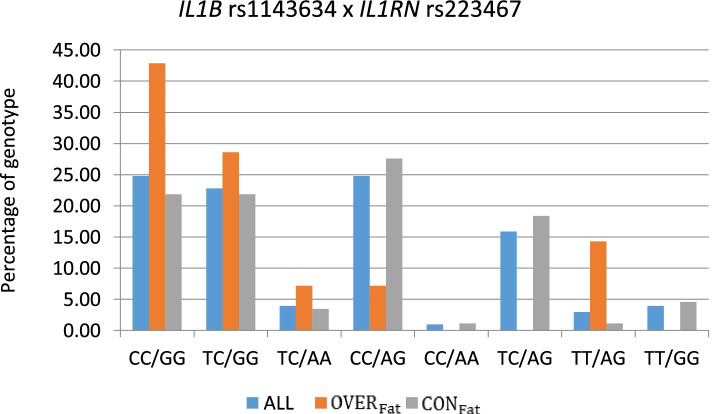
Respective genotype percentages in the examined groups fat %

The most pronounced differences in the frequencies of genotype occurring in either the OVER_BMI_ and CON_BMI_ or OVER_Fat_ and CON_Fat_ groups were observed for the CC/GG genotype (*IL1B* rs1143634/ *IL1RN* rs2234677), which occurred in 40% of the OVER_BMI_ group and 42.86% of the OVER_Fat_ group and in 19,74% of the CON_BMI_ group and 21.84% of the CON_Fat_ group. In contrast, the CC/AG genotype (*IL1B* rs1143634/ *IL1RN* rs2234677) occurred only in 4% of the OVER_BMI_ group and 7.14% of the OVER_Fat_ group and 31.59% of the CON_BMI_ group and 27.59% of the CON_Fat_ group (Figs. 1 and 2).

## Discussion

Obesity is characterized by an abnormal increase in the total amount of triglyceride stored in adipose tissue, resulting from a chronic positive imbalance between energy intake and expenditure [24, 25]. In clinical practice, it is the most often measured as body mass index (BMI), which gives a surrogate measure of overall obesity. According to World Health Organization (WHO) recommendations, body mass index (BMI) higher than 25 is interpreted as indicating overweight, and BMI over 30 indicates obesity [1]; however, it still can underestimate adiposity.

Our study involved the unique, homogenous groups. All participants were students of Military University, so the environmental factors, including the diet, daily schedules, and activities have been the same for several months before the study was conducted. The only factors that distinguished the students for groups were overweighting parameters: BMI (although not the most accurate measurement, but still widely used) and fat %, representing better measure of adiposity. This design of the study allows to avoid the environmental influence and seems the best for the investigation of genetic background of overweighting. The OVER_BM_ group was bigger than OVER_Fat_ which might have been interpreted as suggestion that higher BMI resulted from higher muscle mass. Such phenomenon can be observed in the athletes practicing certain disciplines, but it also results in a very low value of fat%. None of the soldiers examined in our study had high BMI and very low fat%, so both parameters indicated overweighting due to fat content, BMI can understood as more general and fat% as more precise measure of adiposity.

Family studies have previously estimated parent–offspring and sibling correlations in agreement with heritabilities with BMI of 20 to 45% [26, 27]. Then, genome-wide association studies (GWAS) allowed to identify to date approximately 140 obesity susceptibility genes, proved to be associated with measures of adiposity (BMI, fat percentage, and/or waist circumference) [28]. The recent GWAS meta-analysis identified 97 BMI-associated loci (including 56 novel ones) in a study involving 339 224 European adults, accounting for 2.7% of BMI variation [29].

About 30 years ago, obesity was introduced in relation with the expression of proinflammatory cytokines. Hotamisligil et al. [30] made the seminal observation that the expression of mRNA for proinflammatory cytokine tumor necrosis factor alpha (TNFα) was high in adipose tissue in several rodent models of obesity, and when TNFα was neutralized, insulin effect was enhanced. Over the years, a close relationship between metabolic pathways and inflammation was identified, including the role of macrophages and adipocytes which secrete many adipocytokines, acting as messenger molecules. Adipocytokines include inflammatory cytokines, such as IL-1 family, IL-6, IL-8, IL-12, interferons (IFNs), TNFα, and transforming growth factor beta (TGFβ), as well as leukemia inhibitory factor (LIF), monocyte chemoattractant protein (MCP-1), macrophage inflammatory protein (MIP-1), leptin, and resistin [31]. IL-1β has been indicated as the prominent cytokine affecting adipocyte metabolic genes and promoting inflammation and metabolic dysfunction in human adipose tissue [32]. The IL-1 family (except IL-18 and IL-33) is encoded as a cluster and polymorphisms of *IL1A* and *IL1B* genes have been described as related to obesity, however, conflicting results have been reported [22, 23, 33, 34].

In the studies regarding *IL1A*, polymorphism -889 C > T (rs1800587) has been investigated the most frequently. Um et al. (2011) revealed that two *IL1A* polymorphisms -889 C > T (rs1800587) and + 4845 G > T (rs17561) were associated with an increase in BMI in Korean women and lower values were noted in rare allele T carriers. Surprisingly, although fat mass, increased proportionally to BMI, the percentage of body fat was not associated with investigated polymorphisms [23]. The effect of rs1800587 on the transcriptional activity of *IL1A* was additionally tested in vitro using pre-adipocyte 3 T3-L1 cells and in vivo in obese C57BL/6 J mice and the ones injected with IL-1α. The results indicated that *IL1A* -889C > T (rs1800587) was a functional polymorphism of *IL1A* associated with obesity [23, 35]. In Mexican population of adolescents, the same polymorphisms in the *IL1A* gene, constituting haplotype rs17561T-rs1800587T were associated with slight increase in BMI values in males but not females [33]. However, other study, involving males with ischemic heart disease in Western Australia revealed no interaction between *IL1A* rs1800587 polymorphism and BMI, although waist circumference, posing a measure of visceral fat mass was larger in TT carriers [22].

Some studies have reported also *IL1B* polymorphisms as related to obesity. The analysis of + 3953 C/T polymorphism revealed that the T-allele frequency was significantly lower in the overweight (BMI 25–29.9) Korean women and Caucasian men in Sweden [34, 36]. In another Swedish study that involved the population of older men it has been found that *IL1B* − 31 T > C SNP but not *IL1B* + 3953 C > T was associated with total fat [37]. In contrast, in Mexican study, none of the examined *IL1B polymorphisms*: rs1143634 (+3954C > T), rs1143627 (–31C > T), and rs16944 (–511 T > C) were related to BMI [33]. Another pattern has been reported in the population with ischemic heart disease in Western Australia, where *IL1B* + 3954 T > C (rs1143634) SNPs were studied and TT homozygotes had larger waist circumference and the largest value was noted in individuals with two copies of the *IL1A:IL1B* T:T haplotype [22].

Polymorphisms of IL-1 receptor family have been reported as either related [11, 34] or not related (rs419598 and rs2234663) to BMI [33, 37].

In our study, the analyzed polymorphisms: *IL1A* -889C > T (rs1800587), *IL1B* + 3954 T > C (rs1143634), and *IL1RN* -87G > A (rs2234677) alone were related neither to BMI value nor fat %. It might have been surprising taking into account the results of the studies presented above, particularly in regard to *IL1B* + 3953 (rs1143634) that has been suspected to be responsible for body composition in various populations [34, 36]. Although Strandberg et al. (2006) postulated that the association between IL-1 and body fat regulation in humans is robust and not substantially affected by ethnicity, gender, or age [34], it seems more complicated and the results of available studies are at least partially dependent on population parameters. When investigating the relation between genetic background and obesity measurements (including BMI and fat mass), it must be taken into consideration that polymorphisms pose the constant traits and do not change during life, but BMI and fat % may change and depend on many factors including hormonal changes, environmental factors, and individuals’ habits. Thus, if group selection is based on obesity measurements, homogenicity of population is crucial.

Our study revealed the association that according to the authors’ knowledge has not been tested previously. It is currently widely accepted that the interaction between genes, known as epistasis, is the crucial aspect of genetic architecture of complex multicausal phenotypes, both quantitative and qualitative [38–41]. It can explain the phenotype distributions which could not result from the effect of dominant and recessive gene activity,

Taking the above into consideration, apart from the analysis of polymorphism variants for each of the three genes, in our study, the analysis of the interaction of nonallelic genes was also conducted, limited to the two-gene system (*IL1B × IL1RN, IL1B × IL1A, IL1RN × IL1A*) for codominant, dominant, and recessive models. In dominant and codominant models the chance of being overweight (for the combination TC × GG) was much (even 16 times) lower than for the reference combination CC × GG. In addition, the chance of being overweight for genotype combination TC × GG-AG (recessive model) was over 3 times lower in comparison to the reference combination CC × GG-AG, independently of *IL1RN*. These results suggest that the occurrence of rare wild allele T as a heterozygote TC of gene *IL1B* (rs1143634) protected the young men from high BMI. Additionally, association between the SNP-SNP interaction for the *IL1B* rs1143634 x *IL1RN* rs2234677 interaction in the codominant model T/C × G/G (*IL1B × IL1RN*) and C/C × A/G (*IL1B × IL1RN*) and the recessive model T/C × G/G-A/G were related also to the fat % in these young males.

In the codominant model, the odds of being OVER_Fat_ for T/C x G/G (*ILB x IL1RN*) was over 7 times lower than for reference combination C/C x G/G (*ILB x ILRN*); additionally, the odds of being OVER_Fat_ for C/C x A/G (*ILB x ILRN*) was 1.5 times lower than for C/C x G/G (*ILB x ILRN*). In the recessive model, the odds of being OVER_Fat_ for T/C x G/G-A/G (*ILB x IL1RN*) was 10 times lower than for reference combination C/C x G/G-A/G (*IL1B x IL1RN*).

The mechanisms by which IL-1 family cytokines affect BMI or fat % are not completely understood yet. It is widely accepted that obesity is characterized by a variety of cytokine-mediated inflammatory responses of chronic, low-grade nature, which result in metabolic and immune derangement [7, 11, 12]. Among IL-1 family, the role of IL-1β has been found pivotal [42]. Over 15 years ago, it was stated that IL-1β was released by human adipocytes and was regulated by TNFα in obesity. Increased concentrations of IL-1β and TNFα synergistically affect lipid metabolism by the regulation of leptin production and release in adipose tissue [36]. Later hypothesis indicated also that IL-1β mediate regulation of leptin effects at hypothalamic level [34].

Currently, it is widely believed that IL-1β production is strictly related to the transcription and subsequent storage of inactive pro–IL-1β, which is converted to an active form by caspase-1 [7, 12, 42, 43]. Active IL-1β downregulates energy generation and expenditure in adipocyte mitochondria leading to fat accumulation and weight gain [44]. The mechanism of this process involves affecting mitochondrial oxidative phosphorylation by suppressing respiratory chain supercomplex formation, without disturbances in other mitochondrial functions. Supercomplexes formation allows to maintain proton gradient necessary for energy production and so that oxygen consumption and fatty acid oxidation are diminished in IL-1β stimulated cells. The pathway involves activation of IL-1R by IL-1β and further recruitment of MyD88. IL-1 signals are propagated downstream of IL-1R through Myddosome, comprising MyD88 and the IL-1R-associated kinases (IRAK). Recently, the pivotal role of IRAK 2, due to its location within mitochondria has been indicated [45], posing the critical link in the modulation of mitochondrial energy metabolism by IL-1β.

The role of IL-1 family polymorphisms in obesity remains poorly understood and only scanty discussed, mostly in the context of increased cytokine production by the carriers of certain alleles [4, 23, 36, 37]. However, it frequently remains unclear which promoters are more potent. For example *IL1B* − 31 T > C polymorphism has been found important but it was not clear if T or C allele was more potent promoter of IL-1β production and complex interactions with other polymorphisms such as the *IL1B* − 511 C > T SNP have been suggested [37]. Other studies suggested the association between the *IL1B* + 3953C and the IL1RN*2 alleles to produce increased IL-1 bioactivity [34].

The main limitations of our study were the small number of analyzed polymorphisms, lack of AA genotype in IL1A polymorphism rs1800587, and lack of IL-1 measurements in blood and adipose tissue. Since the study involved healthy volunteers recruited from the students of Military University, only minimally invasive procedures were possible and so that even blood could not be taken. However, in order to further confirm the interaction between *IL1B* and *IL-1RN* which has been found in this study, more polymorphisms and larger population should be analyzed.

## Conclusions

This study further confirmed that IL-1 family genes are involved in genetic background of obesity. Our results indicated that even if certain polymorphisms in single genes of IL-1 family cannot be defined as related to obesity in studied population, the interaction should be analyzed. We have found that *IL1B* rs1143634/*IL1RN* rs2234677 interaction for both dominant model and codominant model TC × GG was significant for increased BMI. In this interaction, rare wild allele T as a heterozygote TC of gene *IL1B* (rs1143634) protected the young men from high BMI. Similarly, the SNP-SNP interaction for the *IL1B x IL1RN* in the codominant model T/C × G/G and the recessive model T/C × G/G-A/G were related to the fat %.

Thus, the association studies may help to establish potential new genetic biomarkers for clinical molecular diagnosis of obesity before its metabolic implications

## Materials and methods

### Study protocol

This case-control association study included 101 volunteers—healthy male cadets 19–25 years old. They were randomly recruited via advertising on the Military University campus. The groups did not differ in sex (males only), age, and height (Table 1) as well as also nutrition and activity, as all were students of Military University, lived and ate at the university, and covered the same fitness tests. All participants were selected on the basis of the questionnaire screening for exclusion criteria such as past diseases, injuries, and related ailments and the presence of severe and chronic pain of any organ or system, both in the past and currently. To confirm the good health of the volunteers, general medical examinations were conducted, as well as electrocardiography (ECG).

The examined soldiers lived in a hall of residence on the university’s premises and ate the same meals at the students’ cafeteria. They performed the same physical activity, which is a part of academic syllabus and relates to their military service and responsibilities. The general fitness of soldiers in Polish Army is estimated based on the obligatory fitness test, performed once a year, which is also applied to the Military University students.

Before the study was conducted, participants were acquainted with the protocol and research methods. All participants received a written information sheet concerning the study, providing all pertinent information (purpose, procedures, risks, and benefits of participation). All cadets gave the written consent to participate in the study. The study was conducted in accordance with the Declaration of Helsinki, and the protocol was approved by the Ethics Committee of the Military Institute of Hygiene and Epidemiology—resolution number 07/2018, dated 2.02.2018.

### Anthropometry and body composition

Anthropometric measurements and body composition were obtained using standard methods. Height was measured using a portable stadiometer with a precision of 0.1 cm (without shoes) (TANITA HR-001, Tanita Corporation, Japan). Body composition (including fat %) and body weight were measured using bioelectrical impedance analysis (BIA) performed by the TANITA MC-780 machine (Tanita Corporation, Japan) with accuracy to 0.1 kg according to the procedure specified in the instruction manual (lightly dressed, without shoes). The assessment of BMI values was made in accordance with the criteria set out by WHO [1, 46]

The subjects were divided into two groups depending on their BMI value. The overweight group (OVER_BMI_) was made up of people with BMI of ≥ 25.0, while the control group (CON_BMI_) consisted of people with BMI values between 20.0 and 25.0 [2]. Further analyses were made in groups divided on the basis of sharing fat mass in total subject’s weight (fat %). The control group (CON_Fat_) comprised of subjects with fat % below 20.0% while the overweight group (OVER_Fat_) was characterized with fat % over 20.0% [6].

### Genetic analyses

The buccal cells donated by the subjects were collected using two Copan FLOQSwabs (Interpath, Australia) according to standard procedure. Genomic DNA was extracted from the buccal cells using a High Pure PCR Template Preparation Kit (Roche Diagnostics, Germany). The extraction was performed according to the manufacturer’s instructions. DNA samples of good quality and quantity were stored at − 20 °C for further analysis. The exclusion criteria were as follows: failure in DNA extraction, DNA degradation, abnormal gene detect results, and incomplete basic information.

All samples were genotyped in duplicate on a CFX Connect Real-Time PCR Detection System (BioRad, USA) using TaqMan Pre-Designed SNP Genotyping Assays for *IL1B* (rs1143634) C___9546517_10, *IL1A* (rs1800587) C___9546481_30, and *IL1RN* (rs2234677) C__11948096_10 single-nucleotide polymorphisms (SNPs) (Applied Biosystems, USA), which include primers and fluorescently labeled (VIC and FAM) MGBTM probes for alleles detection. Genotyping was performed according to the manufacturer’s protocol using TaqPath™ ProAmp™ Master Mix (Applied Biosystems, USA). Briefly, conditions for reaction were as following: 30 s of pre-read in 60 °C, 5 min of initial denaturation in 95 °C, cycling 15 s of denaturation in 95 °C, 1 min of primer hybridization and elongation in 60 °C, repeated in 40 cycles, 30 s of final elongation in 60°. The amplified products were visualized and primarily analyzed using CFX Maestro 4.0 Software (BioRad, USA).

### Statistical analysis

IBM SPSS Statistics (version 5.1) was used to calculate differences between groups with Student *t*-test, and genotype frequencies were analyzed using Fisher’s exact test*.* Statistical analysis was done using the SNPassoc package for R (version 1.9-2, R Foundation for Statistics Computing, https://cran.r-project.org). Single-locus analysis including SNP × SNP interaction was performed using SNPassoc (version 1.9.2) considering four genetic models (codominant, dominant, recessive, and overdominant). The models were constructed with respect to the minor allele. The *p*-values for SNP × SNP (epistasis) were calculated using the log-likelihood ratio test. The level of statistical significance was set at *p* < 0.05.

## Data Availability

All datasets used and/or analyzed during whole procedures are available from the corresponding author on reasonable request.

## Abbreviations

IL-1: Interleukin 1
WHO: World Health Organization
BMI: Body mass index
AWCP: Army Weight Control Program
IL-1R: Interleukin 1 receptor
GWAS: Genome-wide association studies
TNFα: Tumor necrosis factor alpha
IFNs: Interferons
TGFβ: Transforming growth factor beta
LIF: Leukemia inhibitory factor
MCP: Monocyte chemoattractant protein
MIP-1: Macrophage inflammatory protein
IL-1Ra: IL-1 receptor antagonist
WHR: Waist to hip ratio
IRAK: IL-1R-associated kinases
SNP: Single-nucleotide polymorphism
IL-1RAcP: IL-1R accessory protein
SIGIRR: Single-Ig IL-1R-related molecule
TIGIRR: Three-Ig-domain-containing IL-1R related
